# Residual feed intake in late-laying hens: immune function, metabolic efficiency, and feed utilization dynamics

**DOI:** 10.3389/fvets.2025.1624978

**Published:** 2025-07-07

**Authors:** Zhouyang Gao, Zhiqiong Mao, Lin Xuan, Guoming Ma, Yan Wu, Guiyun Xu

**Affiliations:** ^1^College of Animal Science and Technology, China Agricultural University, Beijing, China; ^2^Beinongda Technology Co., Ltd., Beijing, China

**Keywords:** residual feed intake, late laying hens, immune function, metabolic function, feed efficiency

## Abstract

The selection of high feed-efficiency animals is essential to address the increasing global demand for animal-derived products while ensuring sustainability. Residual Feed Intake (RFI), a crucial metric in poultry production, enhances feed utilization, optimizes management strategies, and promotes economic viability and environmental stewardship. However, the mechanisms underlying RFI variation remain inadequately understood. This study examined the regulatory pathways of RFI in 70-week-old Rhode Island Red laying hens through comparative analysis of phenotypic parameters, organ characteristics, and serum biochemical profiles between low-RFI (LRFI) and high-RFI (HRFI) groups. The findings demonstrate that RFI functions as a reliable indicator for feed efficiency selection, with LRFI hens demonstrating enhanced immune modulation and metabolic homeostasis while maintaining equivalent egg production performance. These results establish fundamental insights for understanding RFI regulatory mechanisms and developing precision breeding strategies for feed-efficient laying hens.

## 1 Introduction

In livestock and poultry production, feed costs constitute 60–70% of total operational expenditures, with rising feed prices and environmental concerns creating significant challenges to industry sustainability (1). Although China has achieved substantial progress in systematic breeding programs for high-yield laying hens – producing over 300 eggs per hen by 72 weeks of age – significant challenges remain in feed utilization efficiency. The average feed-to-egg ratio of 2.2–2.8:1 remains suboptimal compared to other poultry sectors (broilers, meat ducks), particularly during the late laying phase when metabolic demands increase, resulting in excessive feed waste and reduced profitability (2, 3). Residual Feed Intake (RFI), an internationally recognized metric for individual animal feed efficiency evaluation, has emerged as a vital parameter for optimizing resource allocation (4). Improving RFI-based selection strategies represents a key priority for enhancing feed conversion efficiency and promoting sustainable growth in the poultry industry.

RFI is quantitatively defined as the difference between actual feed consumption and predicted intake based on metabolic body weight, body weight gain, and egg production output, functioning as a heritable biomarker of metabolic divergence in poultry (5). This metric effectively measures species-specific energy requirements for maintenance and growth while remaining phenotypically independent of economically valuable traits. Studies in Korat chickens demonstrate minimal correlations between RFI and meat quality parameters (6, 7). In layer breeding systems, selection for low-RFI hens improves feed conversion efficiency without compromising egg quality or growth performance, while reducing environmental nitrogen excretion by 8–12% and operational costs by 15–18% per production cycle (8). These characteristics establish RFI as an effective selection criterion for sustainable genetic improvement in feed utilization efficiency.

The regulation of RFI represents a complex biological process controlled by coordinated interactions among feeding behavior, nutrient assimilation efficiency, and metabolic pathway dynamics (9, 10). Quantitative genetic analyses indicate moderate to high heritability estimates (h^2^ = 0.25–0.45 across poultry species), with genome-wide association studies identifying 12–18 candidate genes associated with RFI variation in layers (11). Selection programs utilizing RFI optimization demonstrate substantial reductions in broiler feed intake and abdominal fat deposition while maintaining carcass yield and intramuscular fat content (12). Comparative organometrics reveal lower liver mass in low-RFI hens, indicating enhanced basal metabolic rates and energy partitioning efficiency (13). Negative selection for RFI has been successfully implemented across multiple livestock species, consistently achieving feed cost reductions while preserving growth trajectory integrity (14). These multispecies validations confirm RFI’s effectiveness as a selection index for improving feed efficiency in late-laying phase hens through metabolic optimization rather than production trait compromises.

This study aims to examine differences in growth performance, organ characteristics, and serum biochemical parameters between hens with high-RFI (HRFI) and low-RFI (LRFI) during the late laying phase, thereby establishing reference values for comprehensive understanding of RFI regulatory mechanisms and the breeding selection of high feed efficiency laying hen varieties.

## 2 Materials and methods

### 2.1 Birds, housing, diets and experiment groups

The experimental hens were derived from a Rhode Island Red pure-line population provided by China Beinongda Technology Co., Ltd. All subjects were individually housed in separate cages under standardized environmental conditions with ad libitum access to feed and water. The basal diet was formulated according to National Research Council (NRC) guidelines (Table 1). All hens maintained optimal health status throughout the trial, which was conducted in strict compliance with protocols approved by the Animal Welfare Committee of China Agricultural University (Approval AW62801202-1-1). After sorting in ascending order of RFI values, the top 10%/bottom 10% of individuals were selected and categorized into HRFI and LRFI groups (*n* = 6 per group). At trial termination (70-week age), hens were humanely euthanized for biological sampling and subsequent physico-chemical analyses.

**Table 1:** Diet’s composition and nutrient contents (%).

Items	Content
Ingredient	
Corn, %	61.06
Soybean meal, %	23.80
Soybean oil, %	0.80
Wheat bran, %	3.00
Dicalcium phosphate, %	0.90
Stone powder, %	9.00
Salt, %	0.40
Antioxidant, %	0.04
Antifungal agent, %	0.05
Premix^1^, %	0.05
Total	100
Nutrient composition	
AME (MJ/kg)	11.13
Crude protein, %	16.13
Methionine, %	0.36
Lysine, %	0.87
Calcium, %	3.71
Phosphorus, %	0.58

1 Premix: Vitamin premix provided/kg of diet: vitamin A, 10,000 IU; vitamin D_3,_ 3,000 IU; vitamin E, 30 IU; vitamin K_3_, 4 mg; vitamin B_1_, 3 mg; vitamin B_2_, 8 mg; vitamin B_6_, 6 mg, vitamin B_12_, 0.03 mg, niacin, 40 mg, folic acid, 2 mg, biotin, 0.3 mg, pantothenic acid, 18 mg. Mineral premix provided/kg of diet: Mn 100 mg, zinc 100 mg, Fe 65 mg, Cu 9 mg, I 1 mg, Se 0.3 mg.

### 2.2 Sample collection and analyses

#### 2.2.1 Measurement of production and feed efficiency traits

During weeks 67–69 of age, RFI and production traits were measured in this investigation. The evaluated parameters included body weight (BW), body weight gain (BWG), egg number (EN), egg mass (EM), and feed intake (FI). The following measurement protocols were implemented:

BW: Initial and final BW measurements were obtained using a digital platform scale (0.1 g precision), with the average value calculated as metabolic body weight (MBW).
BWG: This parameter was determined by subtracting initial BW from final BW, with daily BWG calculated by dividing the total gain by the 21-day experimental period.
Egg Production: Individual EN was systematically documented throughout the experimental duration.
EM: Daily measurements for each hen were recorded using a precision electronic balance (0.01 g accuracy), with values averaged to establish mean egg weight (EW).
FI: Individual intake monitoring utilized partitioned feeding troughs with metal baffles. Daily feed provision was weighed, with daily feed intake (DFI) derived from 21-day cumulative consumption.

$$\mathrm{RFI}=\mathrm{TFI}-(b_{0}+b_{1}\mathrm{MBW}+b_{2}\mathrm{DEM}+b_{3}\mathrm{DWG}).$$

where TFI represents intake from 69 to 71 weeks of age, b_0_ denotes the intercept, and b_1_, b_2_ and b_3_ represent partial regression coefficients.

#### 2.2.2 Organ indices quantification

After euthanasia through jugular exsanguination, hepatic and splenic tissues were carefully extracted. Adherent adipose tissue was removed from organ surfaces, and excess blood was eliminated through filter paper blotting before gravimetric measurement. Organ indices were determined using the standardized formula:


Organ index(%)=(fresh tissue weight/live body weight)×100

#### 2.2.3 Blood sampling collection

Blood samples were collected from the brachial vein at 70 weeks of age using sterile vacutainers without anticoagulants. The samples were centrifuged at 3,000 rpm for 10 min (4°C) to obtain serum separation. The separated serum was transferred to pre-chilled aseptic cryovials and stored at −20°C for subsequent biochemical analysis.

#### 2.2.4 Serum biochemical profiling

Beijing Jinhai Keyu Biotechnology Co., Ltd. conducted serum analysis using an automated biochemistry analyzer (Kehua ZY KHB-1280) to measure seven metabolic indicators: total protein (TP), albumin (ALB), globulin (GLO), blood urea nitrogen (BUN), creatinine (CRE), uric acid (UA), and lactate dehydrogenase (LDH).

#### 2.2.5 Serum immunological profiling

ELISA kits (Beijing Jinhai Keyu Biotechnology Co., Ltd.) were utilized to quantify serum immune parameters including: Pro-inflammatory cytokines: Interferon-*γ* (IFN-γ), Tumor Necrosis Factor-*α* (TNF-α), Interleukin-1β (IL-1β), Anti-inflammatory cytokines: IL-4, IL-6, IL-10, Endotoxin: Lipopolysaccharide (LPS), Immunoglobulin isotypes (IgA, IgG, IgM) were analyzed.

#### 2.2.6 Serum Metabolic profiling

Circulating concentrations of insulin (INS), adiponectin (ADP), and insulin-like growth factor-1 (IGF-1) were measured using commercial reagent kits (Beijing Jinhai Keyu Biotechnology Co., Ltd.).

### 2.3 Data analysis

Data analysis was performed using Microsoft Excel 2019, and statistical evaluation was conducted through Student’s t-test using SPSS 23.0 (IBM Corp.). Results are presented as mean ± standard error of the mean (SEM). Statistical significance levels were defined as follows: non-significant (*p* > 0.05), significant (*p* < 0.05), and highly significant (*p* < 0.01).

## 3 Results

### 3.1 Production performance differences between LRFI and HRFI groups

As shown in Table 2, total egg production (310.17 vs. 310.33 eggs) and terminal body weight (3046.83 vs. 3010.17 g) showed no significant differences (*p* > 0.05) between LRFI and HRFI groups at 70 weeks of age. However, RFI values demonstrated significant divergence (−105.00 vs. 99.83 g/d, *p* < 0.001), establishing RFI as the primary differentiation factor while maintaining comparable egg production and terminal body weight conditions.

**Table 2:** Comparison of phenotypic data of hens in late egg production with RFI.

Traits^1^	LRFI	HRFI	SEM	*p*-value
EN_70_	310.17	310.33	1.462	0.912
BW_70_	3046.83	3010.17	27.130	0.224
RFI_67-69_ (g/d)	−105.00^b^	99.83^a^	5.449	<0.001

1 EN_70_, Total egg number at 70 weeks of age; BW_70_, Body weight at 70 weeks of age; RFI_67-69_, Residual feed intake from 67 to 69 weeks of age. Values are presented as the mean and SEM (*n* = 6, 70 weeks of age), and different letters indicate significant differences.

### 3.2 Visceral organ index variations between LRFI and HRFI groups

As demonstrated in Table 3, HRFI hens displayed significantly higher hepatic indices compared to LRFI hens (1.26% vs. 1.05%, *p* < 0.05), whereas splenic indices showed no statistical difference between the groups (0.08% vs. 0.08%, *p* > 0.05).

**Table 3:** Comparison of organ index of hens in late egg-laying period with RFI.

Traits^1^	LRFI	HRFI	SEM	*p*-value
Liver index, %	1.05^b^	1.26^a^	0.032	0.001
Spleen, %	0.08	0.08	0.006	0.479

1 Values are presented as the mean and SEM (*n* = 6, 70 weeks of age), and different letters indicate significant differences.

### 3.3 Serum biochemical profile comparisons between LRFI and HRFI groups

Table 4 shows no significant differences between groups (*p* > 0.05) in serum biochemical parameters, including TP, ALB, GLO, BUN, CRE, UA, and LDH.

**Table 4:** Comparison of serum biochemical indices in late egg-laying period with RFI.

Traits^1^	LRFI	HRFI	SEM	*p*-value
TP, g/L	51.08	51.24	1.736	0.926
ALB, g/L	36.45	35.55	3.018	0.771
GLO, g/L	24.95	24.08	2.060	0.682
BUN, mg/L	3.13	3.11	0.339	0.946
CRE, μmol/L	20.27	19.47	0.874	0.379
UA, μmol/L	360.19	364.01	28.591	0.896
LDH, U/L	820.81	803.67	35.112	0.641

1 TP, Total Protein; ALB, Albumin; GLO, Globulin; BUN, Blood Urea Nitrogen; CRE, Creatinine; UA, Uric Acid; LDH, Lactate Dehydrogenase. Values are presented as the mean and SEM (*n* = 6, 70 weeks of age).

### 3.4 Differential serum immune profiles between LRFI and HRFI groups

As illustrated in Table 5, LRFI hens exhibited significantly higher IL-4 (171.67 vs. 140.69 pg./mL) and IgG concentrations (10.17 vs. 7.88 g/L), while displaying lower LPS (34.34 vs. 41.55 U/L) and IL-1β levels (12.69 vs. 16.68 pg./mL) compared to their HRFI counterparts (*p* < 0.05). No significant differences (*p* > 0.05) were detected in IFN-*γ*, TNF-*α*, IL-6, IL-10, IgA, and IgM concentrations between the groups.

**Table 5:** Comparison of serum immunity indices of hens in late egg production with RFI.

Traits^1^	LRFI	HRFI	SEM	*p*-value
IFN-γ, pg./mL	101.84	102.48	5.403	0.908
TNF-α, pg./mL	74.28	78.30	3.435	0.270
LPS, U/L	34.34^b^	41.55^a^	2.114	0.007
IL-4, pg./mL	171.67^a^	140.69^b^	10.006	0.011
IL-6, pg./mL	44.19	44.37	4.102	0.966
IL-10, pg./mL	69.89	72.04	4.195	0.620
IL-1β, pg./mL	12.69^b^	16.68^a^	1.508	0.025
IgA, g/L	1.04	1.05	0.068	0.887
IgG, g/L	10.17^a^	7.88^b^	0.809	0.017
IgM, g/L	0.69	0.74	0.055	0.386

1 Values are presented as the mean and SEM (*n* = 6, 70 weeks of age), and different letters indicate substantial variations.

### 3.5 Serum endocrinometabolic divergences between LRFI and HRFI groups

Table 6 demonstrates significantly higher serum glucose levels in HRFI hens compared to their LRFI counterparts, whereas ADP and IGF-1 concentrations exhibited no significant differences between groups.

**Table 6:** Comparison of serum endocrine metabolism indexes in late laying hens with RFI.

Traits^1^	LRFI	HRFI	SEM	*p*-value
GLU, mmol/L	10.16	11.64	0.514	0.016
ADP, μg/L	18.03	17.09	1.478	0.543
IGF-1, ng/ml	118.16	120.49	6.856	0.741

1 GLU, Glucose; ADP, Adiponectin; IGF-1, Insulin-like Growth Factor 1. Values are presented as the mean and SEM (*n* = 6, 70 weeks of age), and different letters indicate substantial variations.

## 4 Discussion

### 4.1 Impacts of RFI on growth performance, organ indices, and feed efficiency traits in late-laying hens

Feed costs constitute the primary economic factor in poultry production, with RFI functioning as a linear breeding metric whose component trait weights are determined by biological variance (15). Evidence across livestock species [cattle (16), pig (17), poultry (5), ducks (18)] demonstrates that RFI-based selection effectively reduces feed consumption while maintaining production performance and egg/meat quality parameters, indicating its principal influence on FI and feed conversion ratio (FCR). In layer breeding programs where genetic antagonism exists between key selection traits - particularly the negative correlation between egg weight and production quantity - RFI emerges as an effective solution (19). Yuan et al. documented 8–12% FI reduction in low-RFI layers without altered egg production at 37–40 weeks of age (20), while Zhang et al. (21) reported negligible correlation between RFI and daily weight gain in Pekin ducks. The present findings align with these studies, demonstrating equivalent cumulative egg production between high and low RFI groups despite significant RFI divergence. These results validate low-RFI selection as an effective strategy for enhancing feed efficiency in late-laying hens without compromising egg production or growth performance.

The liver functions as the central hub for nutrient metabolism and storage, with hepatic index (liver-to-body weight ratio) variations reflecting hens’ nutritional utilization efficiency and directly influencing laying performance (22, 23). Elevated hepatic indices typically indicate pathological lipid infiltration and metabolic dysregulation, predisposing to conditions like fatty liver hemorrhagic syndrome (24). The spleen’s immunological functions-including pathogen filtration and leukocyte production-make splenic indices essential biomarkers of disease resistance capacity (25). The data revealed significantly lower hepatic indices in LRFI hens compared to HRFI counterparts, demonstrating improved hepatic metabolic efficiency through reduced lipid deposition and enhanced *β*-oxidation activity.

### 4.2 Immunological impacts of RFI in late-laying hens

Genetic and metabolic determinants of feed efficiency primarily localize to pathways governing carbohydrate/lipid/protein metabolism and ion transport, with substantial immunological cross-talk (26, 27). Enhanced immune competence enables hens to combat pathogens, maintain nutritional homeostasis, and regulate feed intake under environmental stressors (28). Serum analysis revealed distinct immunological profiles between RFI phenotypes: LRFI hens exhibited elevated IL-4 and IgG alongside reduced LPS and IL-1β. Specifically, IL-4 potentiates B-cell differentiation for pathogen-specific antibody production (29), while IgG mediates humoral immunity through bacterial toxin neutralization and immunological memory (30). Reduced LPS levels indicate decreased bacterial endotoxin exposure (31), and lower IL-1β reflects attenuated inflammatory responses via NF-κB pathway regulation (32). This coordinated immunomodulation in LRFI hens - characterized by enhanced adaptive immunity and controlled inflammation-optimizes disease resistance while maintaining metabolic resources for egg production, demonstrating RFI’s dual role in feed efficiency and immunological fitness in aging layers.

### 4.3 Metabolic implications of RFI in late-laying hens

Systemic concentrations of blood metabolites serve as physiological biomarkers for feed efficiency, as demonstrated by the investigation into serum metabolic profiles of RFI-divergent hens (33, 34). The analysis revealed significantly lower circulating glucose levels in LRFI hens compared to HRFI counterparts, paralleling previous observations in ducks where low-RFI individuals exhibited reduced triglycerides (TG) and GLU concentrations (35). Significant positive correlations between RFI and both TG and GLU indicate intrinsic linkages between energy metabolism regulation and feed efficiency (36). This metabolic advantage in LRFI hens likely derives from decreased insulin sensitivity, upregulated hepatic glucokinase expression, and optimized gut microbiota composition, collectively maintaining postprandial glucose homeostasis while minimizing energy dissipation through futile cycling pathways.

## 5 Conclusion

This investigation confirms RFI as an effective selection criterion for improving feed conversion efficiency in laying hens, achieving reduced feed consumption while maintaining egg production and growth performance. Serum biomarker analysis revealed enhanced immunological and metabolic profiles in low-RFI hens. These findings establish a theoretical foundation for breeding feed-efficient layer strains through RFI optimization, supporting sustainable poultry production through improved metabolic homeostasis and prolonged productive longevity.

## Data Availability

The original contributions presented in the study are included in the article/supplementary material, further inquiries can be directed to the corresponding author.
